# Quantitative Analysis of Small Particles Present in Surgical Smoke Generated During Breast Surgery

**DOI:** 10.3390/medicina61081422

**Published:** 2025-08-07

**Authors:** Masatake Hara, Goshi Oda, Kumiko Hayashi, Mio Adachi, Yuichi Kumaki, Toshiyuki Ishiba, Emi Yamaga, Tomoyuki Fujioka, Tsuyoshi Nakagawa, Hiroki Mori, Tomoyuki Aruga

**Affiliations:** 1 Department of Breast Surgery, Institute of Science Tokyo, 1-5-45 Yushima, Bunkyo-ku, Tokyo 113-8510, Japan; 2 Department of Diagnostic Radiology, Institute of Science Tokyo, 1-5-45 Yushima, Bunkyo-ku, Tokyo 113-8510, Japan; 3 Breast Center, Dokkyo Medical University, 880 Kitakobayashi, Mibu-machi, Shimotsuga-gun, Tochigi 321-0293, Japan; 4 Department of Plastic and Reconstructive Surgery, Institute of Science Tokyo, 1-5-45 Yushima, Bunkyo-ku, Tokyo 113-8510, Japan

**Keywords:** surgical smoke, breast surgery, health hazards, particle counter, smoke evacuator, small particle

## Abstract

*Background and Objectives*: Surgical smoke generated by energy devices during surgery contains hazardous substances and poses health risks to staff in the operating room. Exposure to surgical smoke must be reduced to minimize the risk of health hazards. Many studies have evaluated surgical smoke qualitatively, but few have performed quantitative assessment. The aim of this study was to quantify the number of particles generated during various breast surgery procedures. *Materials and Methods*: In this prospective, randomized study, breast surgeries performed at Tokyo Medical and Dental University Hospital (the present Institute of Science Tokyo Hospital) between December 2022 and August 2023 were randomly assigned to two groups: the electrosurgical device group and the electrosurgical device with smoke evacuator group. The number of particles generated by energy devices during surgery was measured using a particle counter. *Results*: Surgical smoke was generated in all procedures. The number of measured particles was significantly less in the electrosurgical device with smoke evacuator group than in the electrosurgical device group during all procedures (all *p* < 0.001). *Conclusions*: All breast surgery procedures produced a significant amount of surgical smoke, which was effectively reduced by using an electrosurgical device with a smoke evacuator. These findings support the routine use of smoke evacuators in breast surgery to reduce occupational exposure to hazardous particles. Implementation of such devices could improve operating room safety and may inform future guidelines and institutional policies regarding surgical smoke management.

## 1. Introduction

Surgical smoke generated by energy devices contains hazardous substances harmful to human health [1], including fragments of living cells [2,3,4,5], blood fragments [6], bacteria [7,8], viruses [9,10,11,12,13], and toxic gases such as carbon monoxide [1,14], toluene [15,16,17], methylpropene [18], and acetaldehyde [19]. Studies have shown that the smoke generated from 1 g of tissue is equivalent in total mutagenicity to that from three to six cigarettes [20], and that the average amount of smoke produced daily in the operating room was equivalent to that from 27 to 30 cigarettes [21]. The adverse health effects of cigarette smoking have been thoroughly demonstrated and are widely recognized in the medical literature [22,23,24,25]. It is beyond doubt that exposure to surgical smoke must be reduced to minimize the risk of health hazards. Although many studies have undertaken qualitative evaluation of surgical smoke, few quantitative assessments have been performed. It has been reported that surgical smoke during open surgery contains significantly higher values of total particulate matter 2.5 (PM_2.5_), PM_2.5_ per hour, and maximum PM_2.5_ per minute compared with laparoscopic surgery [26]. As breast surgery is typically performed as an open surgery, not using endoscopy, exposure to surgical smoke may be substantial; however, few studies have focused on surgical smoke in breast surgery. The aim of this study was to quantify the number of small particles generated during breast surgery and evaluate the effectiveness of using an electrosurgical device with a smoke evacuator as a method of reducing exposure to these particles.

## 2. Materials and Methods

### 2.1. Patients

Airborne particles in surgical smoke were measured during 40 breast surgeries performed at Tokyo Medical and Dental University Hospital (the present Institute of Science Tokyo Hospital) between December 2022 and August 2023. To ensure consistent measurement conditions, all measurements were obtained during breast total mastectomies. Patients were alternately assigned on a one-by-one basis to one of two groups, one in which an electrosurgical device was used (electrosurgical device group), and the other in which an electrosurgical device was used along with a smoke evacuator (electrosurgical device with smoke evacuator group), after informed consent was obtained from the patient. Allocation concealment was not performed, and this study was conducted as an open-label trial.

### 2.2. Devices

An electrosurgical device (FORCE 300; Valleylab, COVIDIEN, Dublin, Ireland) set at 35 watts was used for both CUT and COAG. A VisiClear evacuator device (CONMED, Largo, FL, USA) with an evacuation capacity of 130 L per minute was used in the electrosurgical device with smoke evacuator group.

### 2.3. Measurement

A particle counter (KC-52; RION, Kokubunji, Tokyo, Japan) was used to measure small particles in smoke. Three consecutive measurements were obtained for five seconds each, for a total measurement time of 15 s. Prior to the procedure, the distances from the surgeon’s and the first assistant surgeon’s faces to the surgical site were measured multiple times in the operating room. Based on these measurements, the evacuation port of the particle counter was positioned approximately 40 cm away from the surgical site on the operating side and 60 cm away on the contralateral side, corresponding to the typical distances from the surgical site to the faces of the surgeon and the first assistant surgeon, respectively. The measurement periods coincided with activation of the energy device.

### 2.4. Statistical Analysis

Patients’ clinical backgrounds and the particle measurements were compared between the electrosurgical device and the electrosurgical device with smoke evacuator groups using the Mann–Whitney U test or Fisher’s exact test. Comparisons between the two groups were also performed using the Mann–Whitney U test. Correlation was investigated using Spearman’s rank correlation coefficient. *p*-values < 0.05 were considered to indicate significance. Data are expressed as median (range) or number and range values. All statistical analyses were performed using EZR ver. 1.60 (Jichi Medical University, Saitama Medical Center, Saitama, Japan) [27].

## 3. Results

This study included 40 cases: 20 cases were allocated to the electrosurgical device group and 20 to the electrosurgical device with smoke evacuator group. There were no significant differences between the two groups in age (*p* = 0.21) or body mass index (BMI) (*p* = 0.41). All patients were female. Regarding staging, 17 had stage 0, I, or II, and 3 in both groups had stage III or IV. Eleven patients were hormone-positive and HER2-negative, four were HER2-positive, and five were hormone-negative and HER2-negative in the electrosurgical device group. Ten patients were hormone-positive and HER2-negative, six were HER2-positive, and four were hormone-negative and HER2-negative in the electrosurgical device with smoke evacuator group. There was no significant difference between the two groups (*p* = 0.84). Five patients in the electrosurgical device group and eight in the electrosurgical device with smoke evacuator group underwent preoperative treatment, with no significant difference between the two groups (*p* = 0.50). Regarding the surgical procedures, one patient underwent breast total mastectomy, fourteen underwent breast total mastectomy and sentinel lymph node biopsy, and five underwent breast total mastectomy and axillary lymph node dissection in the electrosurgical device group, while three patients underwent breast total mastectomy, nine underwent breast total mastectomy and sentinel lymph node biopsy, and eight underwent breast total mastectomy and axillary lymph node dissection in the electrosurgical device with smoke evacuator group, with no significant difference between the two groups (*p* = 0.32). The median weight of the specimen was 310 g in the electrosurgical device group and 371 g in the electrosurgical device with smoke evacuator group, with no significant difference between the two groups (*p* = 0.91).

Overall, there was no significant difference in any clinical characteristic between the two groups. The characteristics of both groups are shown in Table 1.

**Figure 1:**
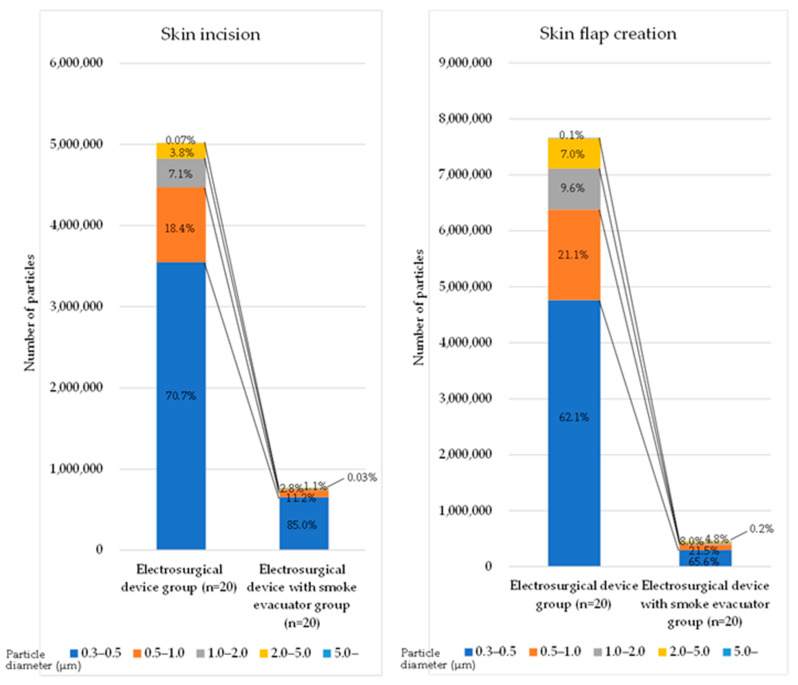

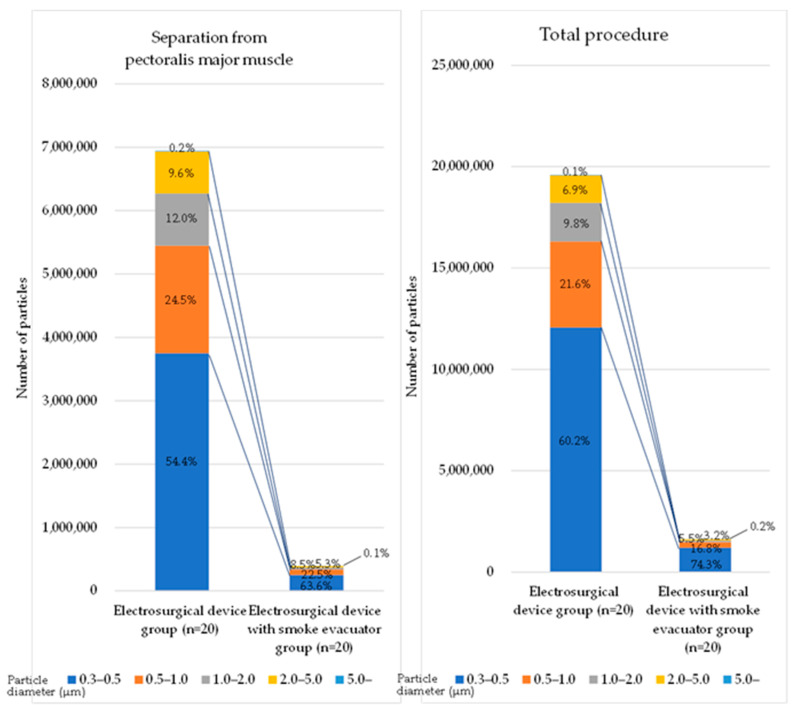
Particle size distribution.

The KC-52 particle counter can measure particles sized 0.3 µm and larger. Figure 1 shows the proportion of each particle size, calculated based on the total number of particles and the number of particles of each size measured during each procedure in each group. In any procedure in both groups, particles in the 0.3–0.5 µm size range accounted for the highest proportion. In all procedures including the total procedures, more than 90% of particles were <2.0 µm in both groups.

**Figure 2:**
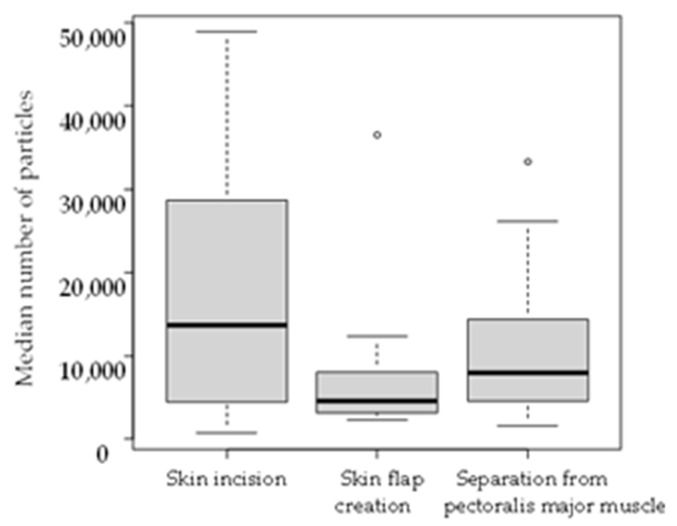
Comparison of median number of particles per measurement among procedures in the electrosurgical device group.

The number of small particles generated during each surgical procedure in the electrosurgical device group was measured. There was significant difference between skin incision and skin flap creation (*p* < 0.05). However, no significant differences were observed between skin incision and separation from pectoralis major muscle (*p* = 0.29) or between skin flap creation and separation from pectoralis major muscle (*p* = 0.072) (Table 2 and Figure 2). In this statistical analysis, no adjustment for multiple comparisons was performed.

Boxplots show the distribution of the average number of particles. The box represents the interquartile range (IQR), which is the range between the first quartile (Q1) and the third quartile (Q3). The line inside the box indicates the median (Q2) of the dataset. The whiskers extend from the box to the minimum and maximum values within 1.5 times the IQR from Q1 and Q3, respectively. Any points outside this range are considered outliers and are plotted individually as points beyond the whiskers.

**Figure 3:**
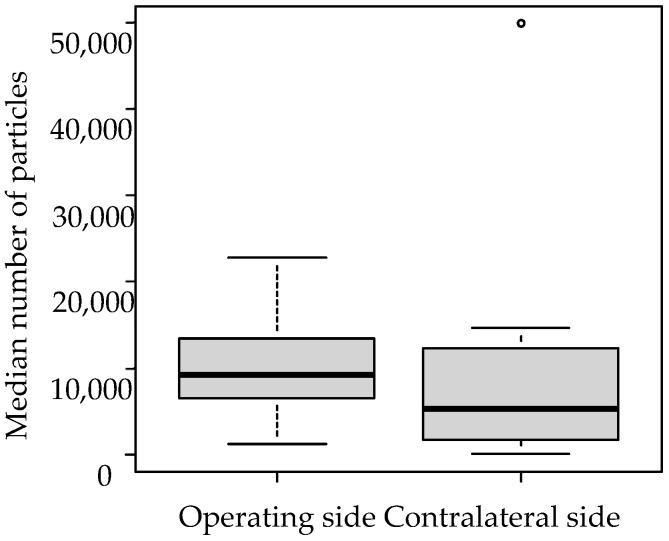
Comparison of the median number of particles per measurement on the operating side and the contralateral side in the electrosurgical device group.

Comparing the median number of particles per measurement on the operating side and the contralateral side in the electrosurgical device group, there were significantly more particles on the operating side (*p* < 0.05) (Table 3 and Figure 3).

**Figure 4:**
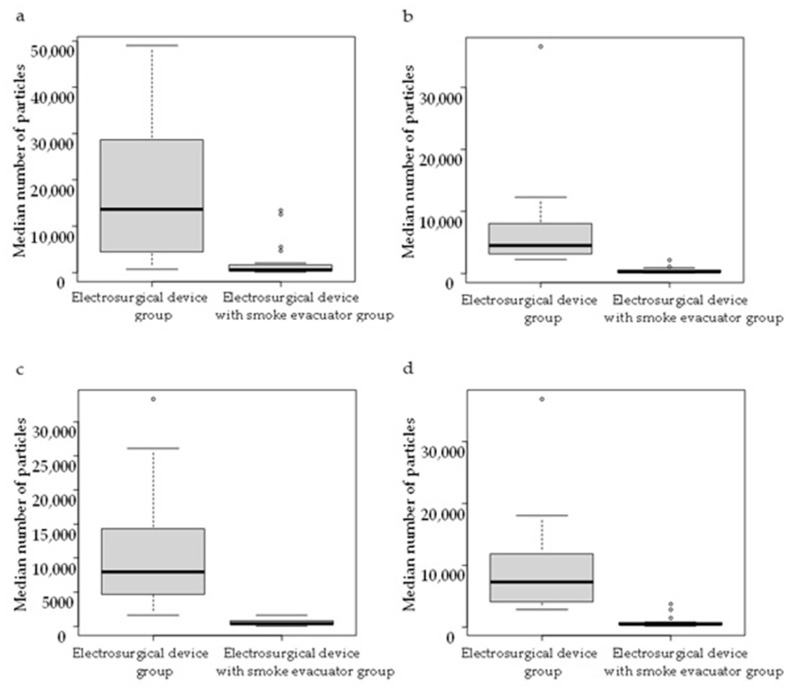
Comparison of the median number of particles per measurement between the two groups for skin incision (**a**), skin flap creation (**b**), separation from pectoralis major muscle (**c**), and total procedure (**d**).

Much surgical smoke was generated in all procedures, and comparing the median number of particles per measurement, there were significantly fewer particles in the electrosurgical device with smoke evacuator group than in the electrosurgical device group during skin incision (*p* < 0.001), skin flap creation (*p* < 0.001), separation from pectoralis major muscle (*p* < 0.001), and the total procedure (*p* < 0.001) (Table 4 and Figure 4).

**Figure 5:**
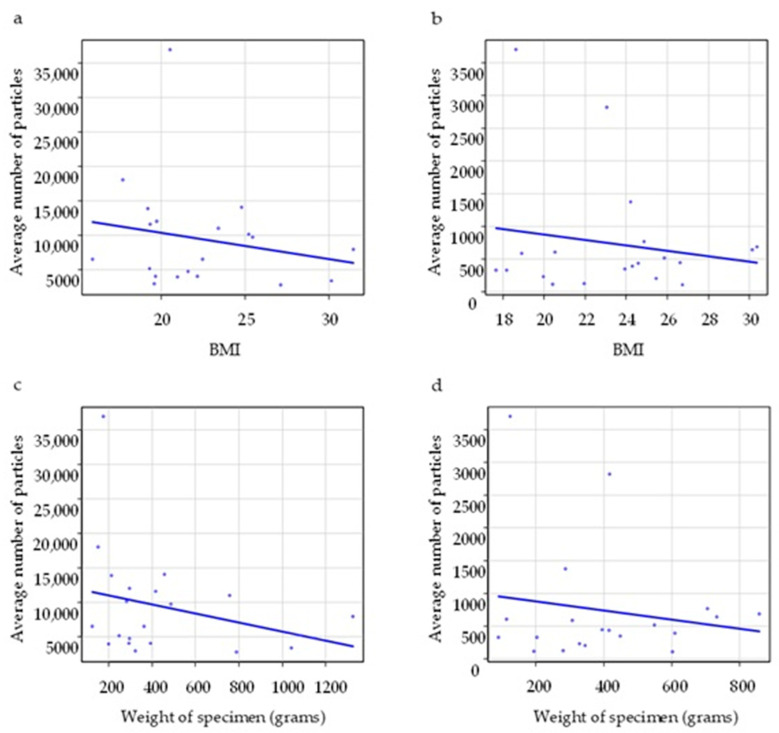
Correlations of the average number of particles with BMI and weight of specimen in the electrosurgical device group (**a**,**c**) and electrosurgical device with smoke evacuator group (**b**,**d**). The horizontal axis of a and b is BMI, and that of c and d is weight of specimen (grams).

There was no significant correlation of the average number of particles with BMI or with weight of the specimen in either group (*p* = 0.31, *p* = 0.64, *p* = 0.26, *p* = 0.44) (Table 5 and Figure 5).

## 4. Discussion

There have been several reports of the dangers of surgical smoke. However, most evaluations have been qualitative, and few quantitative analyses have been conducted. Kameyama et al. [26] reported significantly higher values of total particle matter 2.5 (PM_2.5_), PM_2.5_ per hour, and maximum PM_2.5_ per minute for surgical smoke during open surgery compared with laparoscopic surgery. Okoshi et al. [28] found higher concentrations of PM_2.5_ during breast surgeries than in laparoscopic surgeries. These results suggest that the small particles generated during breast surgery are highly hazardous to human health. To the best of our knowledge, the present study is the first to evaluate surgical smoke exclusively in breast surgery.

The present results show that a substantial amount of surgical smoke was generated in each breast surgery procedure. Up to tens of thousands of small particles were measured during a single test. However, significantly fewer small particles were generated by the electrosurgical device with a smoke evacuator than by the electrosurgical device alone. The median number of particles per measurement during skin incision, skin flap creation, separation from pectoralis major muscle, and for the total procedure was 13,679.7, 4546.0, 7926.9, and 7254.0, respectively, in the electrosurgical device group compared to 576.3, 286.0, 393.8, and 445.9, respectively, in the electrosurgical device with smoke evacuator group. These findings demonstrate the efficacy of the smoke evacuator in reducing the number of small airborne particles in any procedure, thus potentially reducing their harmful health effects.

Comparing the median number of particles per measurement on the operating side and the contralateral side in the electrosurgical device group, there were significantly more particles on the operating side. Although a significant difference was observed, a substantial number of small particles was also detected on the contralateral side. This suggests that surgical smoke poses health risks not only to the operating surgeon but also to the assisting staff. Using an electrosurgical device with smoke evacuator has been shown to effectively mitigate the health risks associated with surgical smoke exposure for assisting staff.

The KC-52 particle counter is capable of counting particles in the 0.3–5.0 μm size range. More than 90% of the particles measured in the present study were sized <2.0 µm. Particles with smaller diameters accounted for a higher proportion of the total particle count. The health hazards associated with PM_2.5_ exposure are widely recognized in the scientific literature [29,30,31,32]. Most of the small particles present in surgical smoke are smaller than PM_2.5_, which raises additional concerns regarding their potential health hazards. Previous studies reported that smaller particles tend to exert more severe health effects [33,34,35]. Regarding the effect of particle size on the respiratory system, Okoshi et al. [36] reported that particles of 0.8–3.0 μm can reach the pulmonary parenchyma. Reducing the number of small particles is therefore crucial for health, with respect to both number and size. The use of an electrosurgical device with smoke evacuator resulted in a marked reduction in particles across all size ranges, demonstrating the effectiveness of the device.

The particles contained in surgical smoke are small in size, and thus, similar to PM_2.5_, are considered to pose health risks such as respiratory diseases. Considering that surgical smoke also contains a substantial proportion of particles smaller than PM_2.5_, the potential harm may be even greater. However, evaluating whether exposure to surgical smoke increases the risk of lung cancer or other diseases is quite difficult due to the need for long-term follow-up and the presence of various confounding factors. Therefore, further investigation is required to assess the cost-effectiveness of implementing smoke evacuators.

In the operating room, not only surgeons but also anesthesiologists, nurses, and other operating room personnel are involved in the procedure, and protecting the health of all staff is of great importance. Smoke evacuators can contribute to this goal. However, there are reports indicating that only approximately 3% of surgeons use smoke evacuators during surgery [21,37], suggesting that awareness of the health risks associated with surgical smoke remains low. In this study, we demonstrated that a substantial number of small particles are generated during breast surgery when using an electrosurgical device and that the use of a smoke evacuator significantly reduced the number of airborne particles. This report highlights the health hazards of surgical smoke and confirms the effectiveness of smoke evacuators, potentially contributing to the development of future guidelines and institutional policies regarding surgical smoke management.

There are various types of smoke evacuators, among which low-cost, self-assembled models are also available. From a cost perspective, it may be meaningful to compare and evaluate their performance against that of commercially available high-performance models. However, we tested the effectiveness of only one type of smoke evacuator due to cost and number of cases in this study. In promoting the widespread adoption of smoke evacuators, factors beyond performance—such as ease of implementation—are also important. Therefore, the use of existing commercial products may offer additional advantages.

This study has several limitations. First, due to their small size, surgical smoke particles are easily affected by airflow, which can cause variation in measurements. To minimize this impact, we standardized the conditions by obtaining measurements specifically during mastectomies and obtained several measurements to allow for the large surgical area. Further research is needed to validate the present data. Second, though the present study showed that significantly fewer particles were generated by the electrosurgical device with a smoke evacuator than by the electrosurgical device alone, the harmful impact of the present measurements on health is unclear. Since PM_2.5_ is an international environmental standard, it may be beneficial to use devices that measure the mass concentration of particles. Third, as no adjustment for multiple comparisons was performed in the analysis of the number of small particles generated during each surgical procedure, the results should be interpreted as reference values in this study.

## 5. Conclusions

The number of particles generated by an electrosurgical device during breast surgery was measured, and all breast surgery procedures produced a significant amount of surgical smoke. Quantitative evaluation of surgical smoke showed that significantly fewer small particles were generated by the electrosurgical device with a smoke evacuator than by the electrosurgical device alone. Occupational exposure to harmful surgical smoke can be reduced by using an electrosurgical device with a smoke evacuator. These findings support the routine use of smoke evacuators in breast surgery to reduce occupational exposure to hazardous particles. Implementation of such devices could improve operating room safety and may inform future guidelines and institutional policies regarding surgical smoke management.

## Figures and Tables

**Table 1:** Patients’ characteristics. Data are presented as median (range) values or the number of patients. Groups were compared using the Mann–Whitney U test or the Fisher’s exact test. Abbreviations: NS, not significant; BMI, body mass index; HR, hormone receptor; HER2, human epidermal growth factor receptor 2; Bt, breast total mastectomy; SLNB, sentinel lymph node biopsy; ALND, axillary lymph node dissection.

	Electrosurgical Device Group (*n* = 20)	Electrosurgical Device with Smoke Evacuator Group (*n* = 20)	*p* Value
Age (y)	60.5 (45–97)	70.5 (40–81)	0.21
Female/male	20/0	20/0	NS
BMI (kg/m^2^)	21.28 (15.90–31.48)	24.08 (17.68–30.37)	0.41
Stage 0–II/III, IV	17/3	17/3	NS
Subtype HR(+), HER2(−) HER2(+) HR(−), HER2(−)	11 4 5	10 6 4	0.84
Preoperative treatment	5	8	0.50
Surgical procedure Bt Bt SLNB Bt ALND	1 14 5	3 9 8	0.32
Weight of specimen (grams)	310 (126–1325)	371 (91–858)	0.91

**Table 2:**
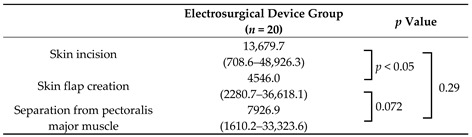
Median number of particles per measurement for each procedure.

Data are presented as median (range) values. Procedures were compared using the Mann–Whitney U test.

**Table 3:** Median number of particles per measurement by side of operation.

	Operating Side	Contralateral Side	*p* Value
Electrosurgical device group (*n* = 20)	9220.9 (1251.9–22,780.1)	5305.5 (69.6–49,788.7)	<0.05

Data are presented as median (range) values. Sides were compared using the Mann–Whitney U test.

**Table 4:** Median number of particles per measurement.

Procedure	Electrosurgical Device Group (*n* = 20)	Electrosurgical Device with Smoke Evacuator Group (*n* = 20)	*p* Value
Skin incision	13,679.7 (708.6–48,926.3)	576.3 (39.4–13,345.9)	<0.001
Skin flap creation	4546.0 (2280.7–36,618.1)	286.0 (25.4–2162.3)	<0.001
Separation from pectoralis major muscle	7926.9 (1610.2–33,323.6)	393.8 (20.6–1601.5)	<0.001
Total procedure	7254.0 (2749.9–36,923.1)	445.9 (105.6–3701.2)	<0.001

Data are presented as median (range) values. Groups were compared using the Mann–Whitney U test.

**Table 5:** Correlations of the average number of particles with BMI and weight of specimen.

		Correlation Coefficient	*p* Value
BMI	Electrosurgical device group (*n* = 20)	–0.28	0.31
	Electrosurgical device with smoke evacuator group (*n* = 20)	0.11	0.64
Weight of specimen	Electrosurgical device group (*n* = 20)	–0.26	0.26
	Electrosurgical device with smoke evacuator group (*n* = 20)	0.18	0.44

Data were analyzed using the Spearman’s rank correlation coefficient. Abbreviation: BMI, body mass index.

## Data Availability

The data and materials used in this study are available from the corresponding author upon reasonable request.

## Abbreviations

The following abbreviations are used in this manuscript:

NS	Not significant
BMI	Body mass index
HR	Hormone receptor
HER2	Human epidermal growth factor receptor 2
Bt	Breast total mastectomy
SLNB	Sentinel lymph node biopsy
ALND	Axillary lymph node dissection
PM_2.5_	Particulate matter 2.5
